# Implant–Natural Teeth Connection for a Patient with Periodontitis and Malocclusion: A Case Report

**DOI:** 10.3390/diagnostics15060765

**Published:** 2025-03-18

**Authors:** Shogo Ando, Atsutoshi Yoshimura

**Affiliations:** 1 Namiki-dori Dental Clinic, 2-2-1 Toyoda, Minami-ku, Nagoya 457-0841, Japan; shogoro_shogoro@yahoo.co.jp; 2 Department of Periodontology and Endodontology, Nagasaki University Graduate School of Biomedical Sciences, 1-7-1 Sakamoto, Nagasaki 852-8588, Japan

**Keywords:** dental implant, periodontitis, orthodontic treatment, oral surgery, digital radiography, treatment planning, diagnosis

## Abstract

**Background and Clinical Significance**: Dental implants are widely used; however, tooth extraction often results in alveolar bone loss and gingival recession, necessitating bone and connective tissue reconstruction, especially in the esthetic anterior regions. To address these issues, implants are occasionally connected to adjacent teeth, but this remains controversial, as complications (e.g., intrusion of natural teeth) have been observed. This report demonstrates the long-term success of implants replaced after removing maxillary bilateral central incisors and connecting them to lateral incisors with reduced supportive bone due to periodontitis. **Case Presentation**: A 57-year-old woman with root fractures in maxillary bilateral central incisors, periodontitis, and malocclusion was treated with connecting implants and natural teeth. Bone levels surrounding maxillary bilateral lateral incisors were diminished due to root fractures in adjacent central incisors and periodontitis. After initial periodontal therapy, hopeless maxillary central incisors were extracted, replaced with implants using a digitally simulated surgical guide, and guided bone regeneration and connective tissue grafting were performed. Implants were connected to lateral incisors with provisional restorations, and orthodontic treatment was initiated following digital set-ups incorporating implants into the overall strategy. Final porcelain-fused-to-zirconia restorations were placed after orthodontic treatment. At the 5-year follow-up, gingival morphology, coloration, and position of lateral incisors remained stable. **Conclusions**: This case demonstrates that connecting implants to natural teeth in the anterior region can effectively maintain periodontal tissues around natural teeth and allow for minimally invasive, short-term, and esthetic treatment. However, careful long-term observation through maintenance is necessary due to limited evidence for this approach in the anterior region.

## 1. Introduction

Dental implants have become a common treatment option for individuals with missing teeth. However, in the anterior region, these implants must satisfy dual requirements: in addition to their fundamental role as prosthetic replacements, implants are expected to meet high aesthetic standards. The visible nature of this area necessitates that implants restore function and harmonize visually with the surrounding natural dentition. Recently, novel methods, such as socket preservation and partial extraction therapy, have been developed to combat the progressive loss of the alveolar bone after tooth extraction [1,2]. Currently, evidence supporting the efficacy of the first method in reducing long-term crest resorption is insufficient. The second method is known to be technique-sensitive and carries risks of complications, such as ankylosis and perforation of the remaining tooth root.

Connecting implants to natural teeth offers several benefits, including reduced tooth extraction, prevention of bone loss, and preservation of the anterior region’s aesthetic appearance [3,4]. Despite the pros and cons of this approach, the main opposition to it appears to stem from Brånemark’s protocol, which recommends that implants should operate independently [5]. In contrast, Lindh did not rule out the prosthetic option of preserving natural teeth and connecting them to implants [6]. In a consensus report published in 2000, Belser et al. stated that the connection to natural teeth should only be used in exceptional situations [7].

Regarding survival and complication rates, Lang et al. found that the average loss rates for single implant placement and natural teeth–implant connection were 0.94% and 2.1%, respectively, suggesting that implants without connection are preferable [8,9]. In a meta-analysis, La Monaca et al. found no significant differences between single implant placement and natural teeth–implant connection in terms of abutment failure, biological and mechanical complications, prosthetic loss, and prosthetic complications. These findings indicated that a natural teeth–implant connection may be a viable treatment option, particularly when it reduces the technical complexity due to the severe condition of the remaining dentition and treatment costs [10]. A meta-analysis performed by Alsabeeha et al. revealed that the only significant difference between single implant placement and natural teeth–implant connection was a decrease in the peri-implant bone level, with connection linked to less decrease and better hard tissue preservation, indicating that connection is superior in preserving hard tissue [11]. Numerous studies have investigated the connection between natural teeth and implants. However, research specifically focusing on their connection in the anterior dental region is limited to case reports, and systematic reviews have not been conducted thus far [12].

In this report, we describe the 5-year functional and esthetic stability of the periodontal tissues of the surgical site achieved by placing implants after the extraction of the maxillary bilateral central incisors and connecting them to the bilateral lateral incisors. This case was associated with a favorable outcome.

## 2. Case Presentation

A 57-year-old female patient visited the Namiki-dori Dental Clinic (Nagoya, Japan) in January 2016 with a chief complaint of discomfort in the maxillary anterior teeth during mastication. She had porcelain-fused-to-metal restorations set on the maxillary bilateral central incisors at another clinic. Although she became aware of swaying approximately 2 years ago, she ignored the observation due to the absence of pain. She had no history of smoking or systemic disease.

**Figure 1:**
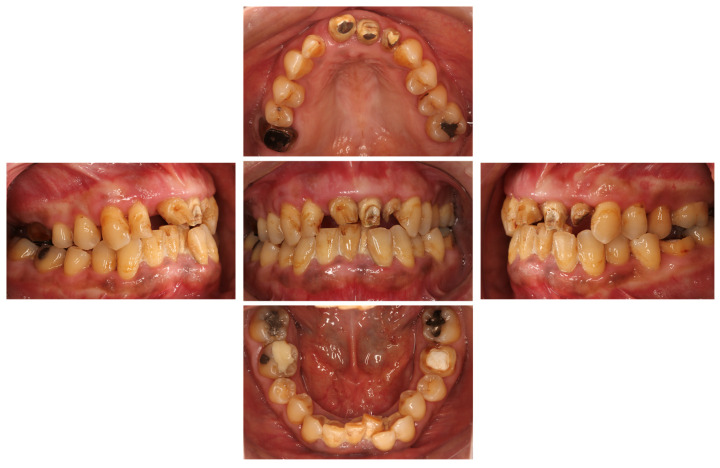
Intraoral photograph at the initial examination.

**Figure 2:**
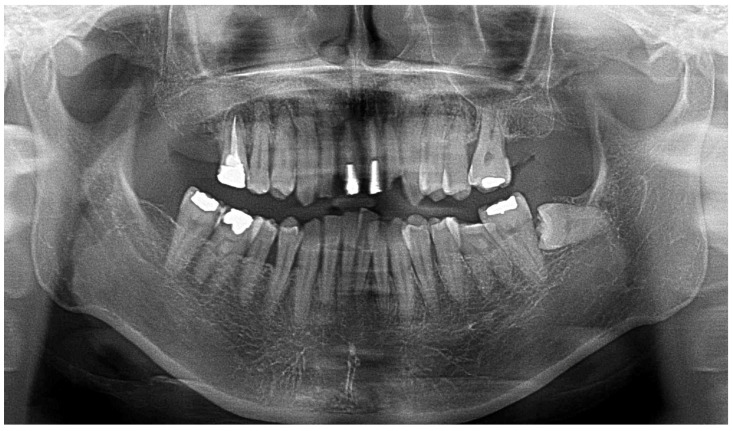
Panoramic radiograph at the initial examination.

**Figure 3:**
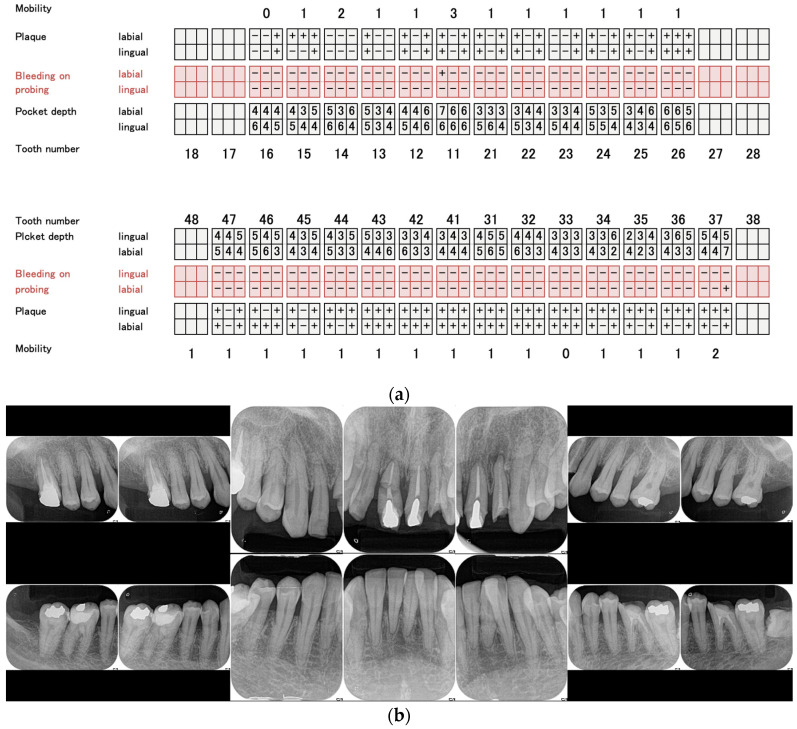
Results of initial periodontal examination (**a**) and dental radiographs (**b**).

At the time of the initial examination, the porcelain-fused-to-metal restorations of the maxillary bilateral central and maxillary left lateral incisors were detached, and the maxillary left lateral incisor was affected by caries (Figure 1). A sinus tract was found on the buccal side of the maxillary right central incisor. Crowding was also observed in her dentition. The maxillary bilateral second molars had been extracted, and the maxillary right first molar and mandibular bilateral first molars were under treatment, resulting in a significant decrease in occlusal support in the molar regions (Figure 1 and Figure 2). Periodontal examination revealed extensive plaque accumulation throughout the dentition, with a plaque control record of 75% (Figure 3a). Periodontal pockets of 6–7 mm and suppuration were observed in the maxillary bilateral central incisors. Radiographs showed radiolucency around the roots of the maxillary bilateral central incisors and a fracture line around the center of the roots (Figure 3b). Marked alveolar bone resorption was observed around multiple teeth, including the maxillary bilateral lateral incisors. Based on the examination results, the patient was diagnosed with bilateral maxillary central incisor root fracture, malocclusion (classified as Angle class 1 with crowding), and stage 4 periodontitis (grade C).

**Figure 4:**
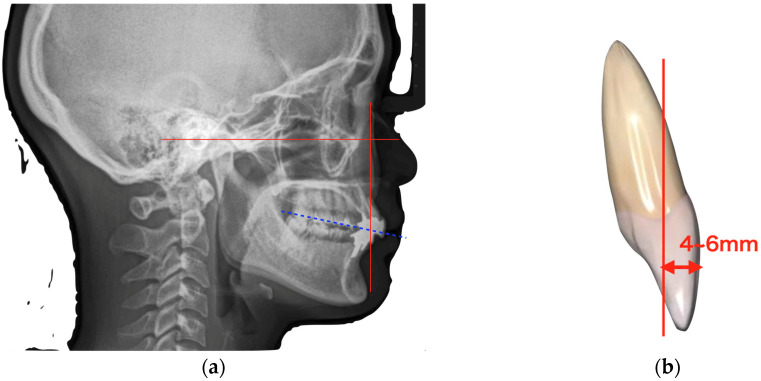
Determination of implant placement position using lateral cephalometry. (**a**) The incisal edge position and the virtual occlusal plane were determined using lateral cephalometry. (**b**) The anterior–posterior positioning of the anterior teeth was determined using the McNamara line (red line).

**Figure 5:**
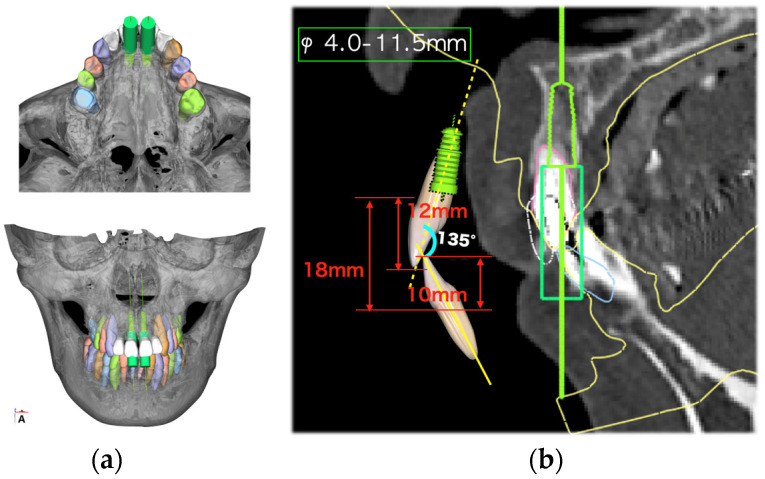
Preoperative digital simulation. Digital setup and wax-up were conducted with reference to the incisal edge position determined by cephalometry (**a**). Implant placement position was determined (**b**).

Regarding the treatment plan, the maxillary bilateral central incisors were judged to be hopeless because of significant bone resorption and root fracture. After explaining the advantages and disadvantages of removable dentures, fixed partial dentures, and implants for bilateral maxillary central incisors that were judged hopeless, the patient preferred implants for the long-term prognosis of bilateral adjacent teeth. If a fixed partial denture is selected, the maxillary bilateral lateral incisors and canines must be drilled. If a removable denture is selected, there are concerns regarding the esthetics and loading of the clasped teeth. However, even if implants are placed after the extraction of the maxillary bilateral central incisors, the bone support of the lateral incisors is reduced, raising concerns about functional stability. The crown-root ratio of the bilateral lateral incisors was less than 1:1, which was expected to worsen with alveolar bone resorption after central incisor extraction. This makes an independent function difficult. Connecting them to implants allows them to remain functional. In addition, buccal gingival recession was expected after extracting the maxillary bilateral central incisors, and the patient wished to undergo esthetic treatment, including orthodontics, due to malocclusion. However, without anterior anchors, reproducing the dental arch for bracket orthodontics is difficult and prolongs the orthodontic period. Digital simulation can predict the final dental arch in advance, thereby allowing for implant placement before orthodontic treatment. Once osseointegrated, these implants can serve as orthodontic anchors, thereby reducing the treatment duration. Therefore, the following treatment plan was made, explained to the patient, and consent was obtained:

1. Determination of the incisal edge position and virtual occlusal plane using lateral cephalometry (Figure 4a);
2. Determination of the anterior–posterior positioning of the anterior teeth using the McNamara line (Figure 4b);
3. Preoperative simulation using digital simulation software (BioNa, version 6.0 Wada Precision Casting Laboratory, Osaka, Japan), digital setup, and digital wax-up to determine the implant placement position using the incisal edge position determined by cephalometry as a reference (Figure 5);
4. Implant placement using a surgical guide to place the implant in the exact position;
5. Bone regeneration with autologous bone and membrane;
6. Connective tissue graft to ensure adequate soft tissue thickness and provisional restoration after the establishment of osseointegration;
7. Orthodontic treatment;
8. Final prosthetics.

**Figure 6:**
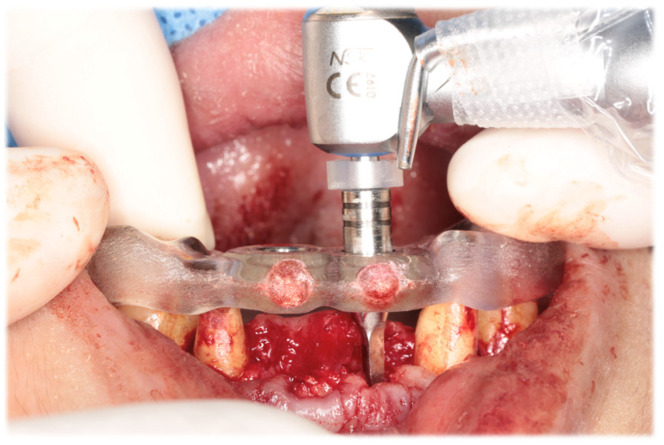
Intraoral photograph during implant placement. A surgical guide was used to place implants in digitally planned locations after extraction of the maxillary bilateral central incisors.

**Figure 7:**
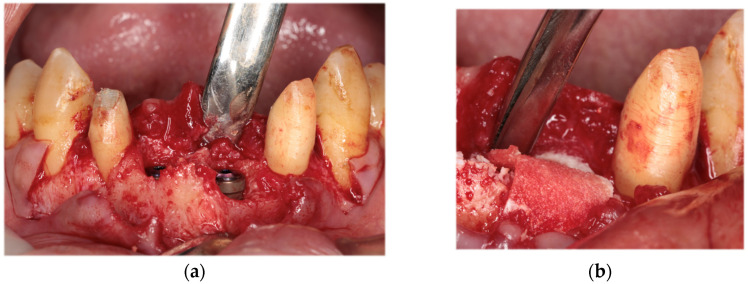
Intraoral photograph during guided bone regeneration (GBR). Following placement of the implant in the simulated position (**a**), GBR was performed using autologous bone and a membrane (**b**).

**Figure 8:**
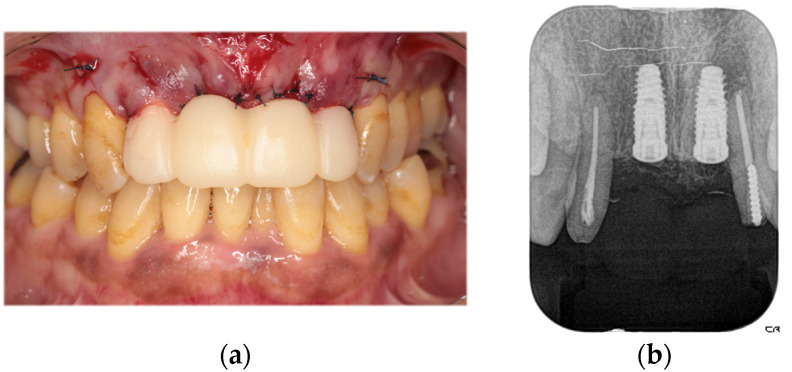
Intraoral photograph (**a**) and dental radiograph (**b**) immediately after implant placement.

Initial periodontal therapy was begun in February 2016. Concurrently, caries treatment, root canal treatment, and interim prostheses were performed on the maxillary right first molar, mandibular bilateral first molars, and mandibular right second molar. Subsequently, full-mouth scaling and root planing was performed, followed by periodontal tissue regeneration therapy on the maxillary bilateral molars. After the inflammation in periodontal tissues subsided and the occlusal support area was broadened with the interim prosthesis, computer-based simulations were used to assess various options for implant positioning. Prior to the implant placement, a precise plan was created by examining and processing cephalometric and computed tomography scans along with data from intraoral scans. The implant (T3 DCD Tapered Implant, 4/3 × 11.5 mm; BIOMET 3i, Palm Beach, FL, USA) was placed in December 2016 using a surgical guide (Figure 6). Autologous bone and a membrane (Osseoguard; BIOMET 3i) were used in the process of guided bone regeneration to address bone defects (Figure 7). The surgical procedure included making periosteal release incisions followed by primary closure of the operative site. Radiographic images revealed that the implants were positioned parallel to each other, although the implant on the left side was close to the adjacent tooth (Figure 8).

**Figure 9:**
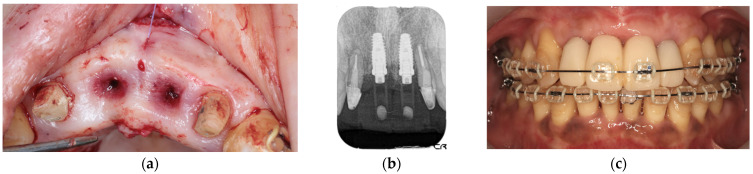
Intraoral photograph during connective tissue grafting (**a**), dental radiograph after provisional restoration placement (**b**), and orthodontic treatment (**c**).

**Figure 10:**
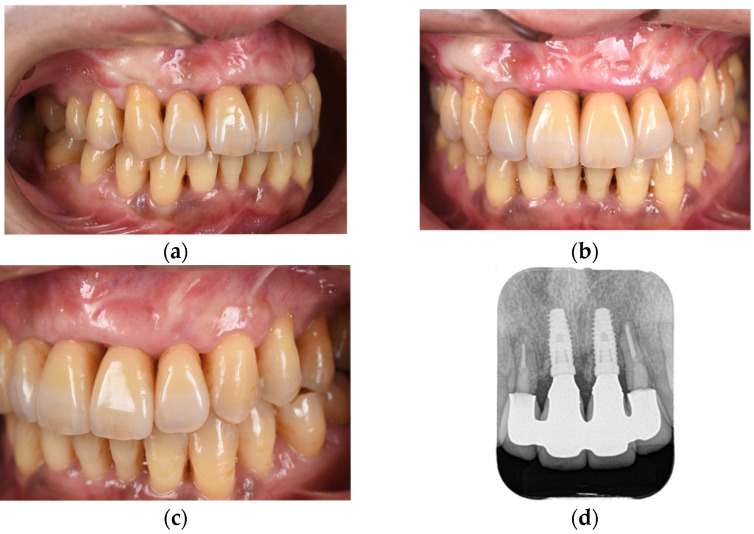
Right-lateral (**a**), frontal (**b**), and left-lateral (**c**) views and dental radiograph (**d**) obtained immediately after the cementation of the final prosthesis.

**Figure 11:**
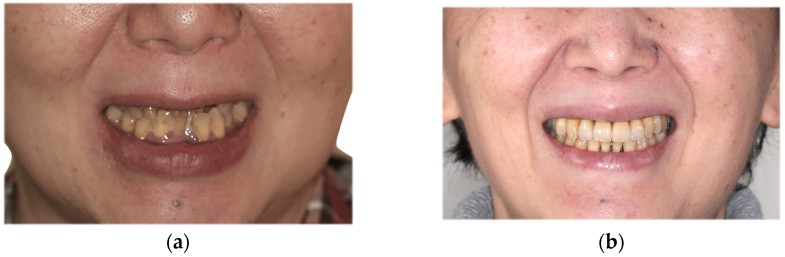
Facial images obtained during the initial examination (**a**) and immediately following the placement of the final prosthesis (**b**).

The second-stage implant exposure procedure was conducted in March 2017. Subsequently, a connective tissue graft was applied to enhance the soft tissue volume and improve aesthetic outcomes (Figure 9a). Provisional restorations were placed (Figure 9b), and orthodontic treatment was initiated (Figure 9c). The orthodontic treatment was completed in February 2018. Although the implant on the left side was in close proximity to the adjacent tooth, no clinical issues were observed around the implant or in the remaining teeth immediately after placement or during the provisional restoration period. Therefore, porcelain-fused-to-zirconia restorations were installed in the month (Panavia; Kuraray Noritake Dental Inc., Niigata, Japan) (Figure 10). Due to the low bone support of the maxillary bilateral lateral incisors and potential complications, screw retention was preferable to allow prosthetic device replacement. However, the structural complexity of the connection with the natural teeth makes screw retention difficult; therefore, the superstructure was cemented. Although provisional cementation would have been ideal for potential reprosthetics, luting cement was used to prevent intrusion of natural teeth. The patient’s oral aesthetics showed notable enhancement since the initial consultation, as depicted in Figure 11.

**Figure 12:**
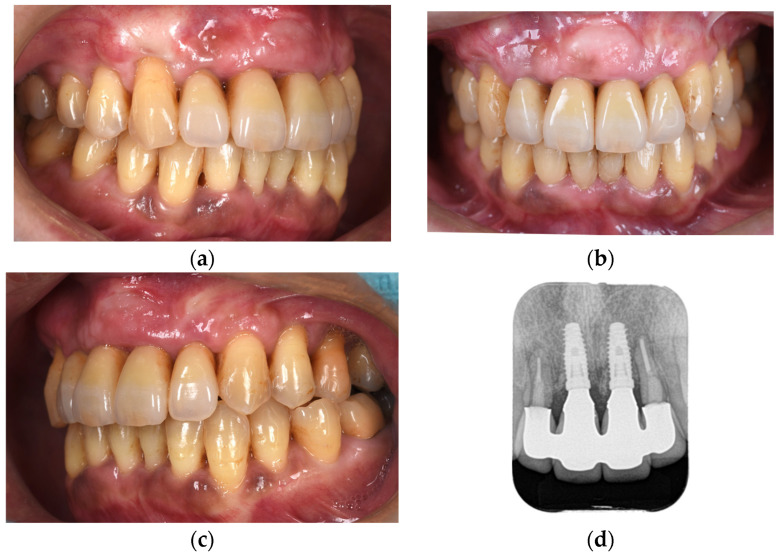
Right-lateral (**a**), frontal (**b**), and left-lateral (**c**) views and dental radiograph (**d**) at 5 years postoperatively.

**Figure 13:**
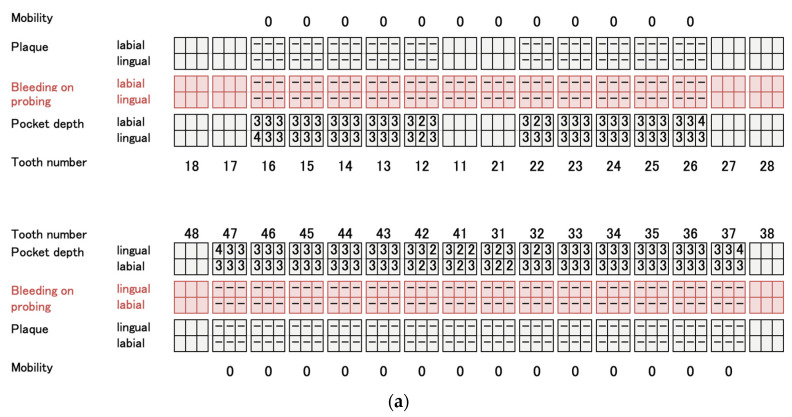

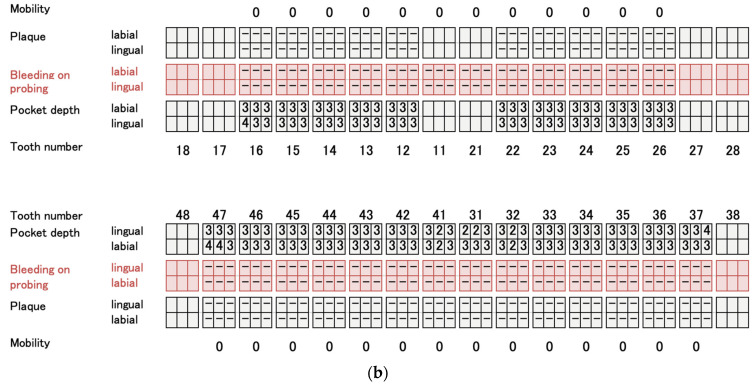
Periodontal examination immediately after placement of the final prosthesis (**a**) and at the 5-year follow-up (**b**).

Following the completion of prosthetic treatment, maintenance appointments were scheduled every 3 months. After a 5-year maintenance period, the gingival tissue showed no significant alterations in shape or color, and the adjacent teeth maintained proper functionality (Figure 12). The periodontal status of the patients changed minimally immediately after the placement of the final prosthesis and at the 5-year follow-up (Figure 13).

## 3. Discussion

The complexity of this case arose from the ongoing controversy regarding the connection between the natural teeth and implants, especially considering the limited documentation of such connections in the anterior region. The connection between the natural teeth and implants in this case prevented resorption of the hard and soft tissues surrounding the lateral incisors. This eliminated the necessity for extensive tissue regeneration procedures, resulting in a less invasive approach and reduced treatment period.

Complications associated with the connection of natural teeth to implants include prosthetic loss, implant loss, natural tooth loss, natural tooth intrusion, and resorption of the crestal bone around the implants. This can be attributed to the structural differences between the natural teeth and implants. Biomechanically, natural teeth and implants differ in the amount of pressure displacement, the relationship between displacement and load, the fulcrum of inclination, and stress distribution, which are attributed to the presence or absence of the periodontal ligament. According to Mamalis et al., when a force of 0.1 N is applied, the movement of natural teeth is 50–200 μm, whereas the movement of implants is <10 μm [13]. The pressure displacement for natural teeth and implants is 25–100 μm and 3–5 μm in the axial direction [14] and 150–200 μm and 17–66 μm in the horizontal direction [15], respectively. Natural teeth and implants respond differently to loads. Implants exhibit linear displacement with increased load, mirroring the bone movement. Natural teeth demonstrate a more complex response: initial nonlinear movement followed by linear displacement alignment with alveolar bone movement [14]. Unlike natural teeth, where the fulcrum of inclination is located in the upper third of the root, implants have a fulcrum near the alveolar ridge [14]. Several hypotheses have been proposed to elucidate the phenomenon of natural tooth intrusion when connected to implant-supported fixed partial dentures. These explanations include differential energy dissipation theory [16], impaired rebound memory theory [17], bending load theory [18], and stress wave theory [19]. Nevertheless, these theories lack complete verification, and additional research is necessary to confirm their validity [19,20]. Despite the common association of these complications with occlusal load, this particular case involved connecting anterior teeth to implants. The occlusal forces on these teeth were less substantial than those experienced by molars, and the implant–tooth interface would have been only marginally affected by such occlusal pressures.

While anterior teeth may be less prone to load-related complications than molars, it is crucial to prevent bacterial-induced bone loss, such as that caused by periodontal disease and peri-implantitis, to ensure a favorable long-term outcome [21]. Compared with molars, front teeth have thinner buccolingual bones, particularly on the buccal aspect, making them more susceptible to loss as periodontal disease progresses [22]. Given these considerations, recall appointments were scheduled at 3-month intervals during the maintenance phase. The dental hygienist closely monitored the progress, while providing detailed instructions to the patient for self-care practices. At the initial consultation, the patient’s plaque control record value was 63%. However, following completion of the initial treatment, this value consistently remained below 20%. Ongoing vigilant monitoring was conducted to detect the onset of gingivitis or peri-implant mucositis, as well as to identify any radiographic alterations in the hard tissues. Despite the presence of periodontal disease during the initial assessment, the patient reported no history of diabetes mellitus or tobacco use. This may explain the lack of signs of bone resorption in the lateral incisors during the 5-year follow-up period. Both hard and soft tissues remained stable postoperatively. From an aesthetic perspective, the long-term prognosis for this patient appears promising. However, individuals with a history of smoking or diabetes should be particularly vigilant regarding the potential for periodontal disease and peri-implantitis. The capacity to mitigate these risks may significantly influence overall prognosis, making it a key factor in long-term oral health outcomes.

The implant was placed and allowed to osseointegrate before the temporary restoration was installed. Thereafter, orthodontic treatment was initiated, with the implants functioning as anchors for orthodontic procedures. Research has demonstrated that orthodontic treatment duration can be reduced by strategically positioning implants before initiating orthodontic procedures. This approach involves utilizing precise preoperative digital wax-ups and integrating implants into orthodontic treatment plans. According to Blasi et al., the treatment duration can be reduced to 8 months by inserting implants prior to orthodontic procedures in cases involving the anterior esthetic zone. Without implants, such treatments typically require 14–16 months for completion [23]. Additionally, minimal to no movement of the lateral incisors was detected when their positions were evaluated 5 years after surgery in comparison with their preoperative locations. The observed outcome can potentially be explained by several factors: the optimal positioning of the implant was predetermined through digital preoperative planning; adequate gingival thickness was achieved by grafting connective tissue onto the anterior maxillary teeth; and the occlusal support area was increased by prosthetic treatment to the molars. Nevertheless, insufficient evidence exists regarding the relationship between natural teeth and implants in the anterior region; therefore, careful monitoring should be maintained during follow-up care.

## 4. Conclusions

When multiple maxillary anterior teeth are absent, connecting implants to the natural teeth can be an effective treatment strategy. This approach preserves the periodontal structures surrounding the remaining natural teeth, while offering a swift, minimally invasive, and aesthetically pleasing solution for patients.

## Data Availability

The original contributions presented in this study are included in the article. Further inquiries can be directed to the corresponding author.
